# Prevalence of Irritable Bowel Syndrome (IBS) in the Arab World: A Systematic Review

**DOI:** 10.7759/cureus.65421

**Published:** 2024-07-26

**Authors:** Omar Almansour

**Affiliations:** 1 Department of Medicine, Qassim University, Qassim, SAU

**Keywords:** middle east, arab world, prevalence, ibs, irritable bowel syndrome

## Abstract

Irritable Bowel Syndrome (IBS) is a common functional pathology of the gastrointestinal tract (GIT) across the globe. The prevalence rate of IBS varies across the regions. In the present systematic review, we aim to investigate the prevalence of IBS in Arab countries in recent years. To identify relevant studies, a comprehensive search was undertaken in various databases including CINAHL (Cumulated Index to Nursing and Allied Health Literature) Ultimate, Scopus, PubMed, and Web of Science. Furthermore, Google Scholar was also explored to identify relevant studies. The inclusion criteria included studies that assessed IBS in the Arab world and were published in the English language. Fifty-two cross-sectional studies from seven countries, encompassing 51,683 participants, were included. The majority of the included studies were conducted in Saudi Arabia (n=40), followed by Jordan (n=4) and Egypt (n=3). The highest prevalence rates were reported by studies from Saudi Arabia, Lebanon, and Jordan. Low prevalence rates (<20%) were noted in 19 studies included in this systematic review. Female predominance in IBS prevalence was observed in most studies, while only two studies indicated higher prevalence in males. Key risk factors included family history, anxiety, depression, gastroesophageal reflux disease, low income, diabetes, low water intake, workload, occupation, food allergy, smoking, age, chronic diseases, and stress. IBS prevalence in the Arab world varies significantly. Female predominance was seen in the present systematic review as well.

## Introduction and background

Irritable bowel syndrome (IBS) is a chronic functional pathology of the gastrointestinal tract (GIT). This disease is characterized by chronic abdominal pain, bloating, and altered bowel habits, which can significantly impair the quality of life [1]. The severity of symptoms varies in different individuals with some experiencing severe disease whereas others experiencing mild symptoms. The pathophysiology of IBS is not completely understood; however, it is believed to be a multifactorial pathology [2,3]. Due to variations in symptoms, the diagnosis of IBS poses challenges. Furthermore, there is a lack of precise biomarkers and sensitivity and specificity testing. Different diagnostic guidelines have been proposed such as Kruis scoring system and Manning and Rome criteria. The Rome I, II, and III criteria have been widely adopted for the diagnosis of IBS. Recently, Rome IV criteria is the most commonly accepted criteria for IBS [4] .

IBS is a multifactorial condition with several risk factors. Previously, studies have recognized both mental and physical factors to influence the development of IBS. For example, stress has been implicated in the progression of the disease [5]. Other well-known risk factors of IBS include chronic inflammation of the intestine, and altered intestinal flora [6,7]. Due to the involvement of various risk factors, the prevalence of IBS is influenced by genetic, environmental, cultural, and dietary factors. Throughout the world, the prevalence of IBS varies considerably. Approximately 10-20% of the population in the world suffers from IBS. Women and individuals aged below the age of 50 years are particularly vulnerable to IBS [8]. Epidemiological studies on IBS in the Arab world are relatively scarce compared to Western countries. However, the available literature suggests that the prevalence of IBS in the Arab world is comparable to, if not higher than, that in Western countries. A systematic review by Almasary et al. reported a pooled prevalence of 20.7% in Saudi Arabia [9]. Their systematic review and meta-analysis included 38 studies and 26,567 participants.

The difference in prevalence in the Arab nations can be due to various factors including lifestyle, diet, and healthcare access. Traditional healthcare practices and varying levels of awareness and education about IBS among healthcare providers and the general population further complicate the epidemiological landscape of IBS in the Arab world. For example, a study by Issa et al. reported that only 50% of the participants were aware of IBS in Saudi Arabia [10]. Healthcare infrastructure and access to medical care vary widely across the Arab world, affecting the diagnosis and management of IBS. In some countries, advanced healthcare facilities and specialized gastrointestinal clinics are available, while others may have limited resources. There has been a continuous rise in the prevalence of IBS in the Arab World. For example, Isbister and Hubler reported in 1998 that the prevalence of IBS is rare in Saudi Arabia [11]. Similarly, a prevalence of 1.35/100 000 person-years was reported from Oman in 1997 [12]. However, a systematic review by Alosaimi et al. reported a prevalence of 8.9-31.8% in the Arab world in 2016 [13].

As IBS can have a significant impact on the quality of life and healthcare systems, it is important to understand the exact prevalence of IBS in the Arab world as currently, there is a paucity of up-to-date research on this. This systematic review aimed to summarize the latest evidence on IBS in the Arab World.

## Review

Search strategy

A systemic search was carried out in various databases to identify the prevalence of IBS in the Arab World. The searched databases included PubMed, Web of Science, CINAHL (Cumulated Index to Nursing and Allied Health Literature) Ultimate, and Scopus. To further increase the relevant literature, Google Scholar was also searched. A combination of keywords was used during the search strategy. Commonly used keywords included Epidemiology,” or “Incidence,” “Irritable bowel syndrome,” or “IBS,” and “Arab World,” or “Middle East”. Apart from these keywords, different alternatives of the keywords were also used. The details of all the keywords are presented in the Appendix A. The inclusion criteria of this systemic review were as follows: (i) studies that investigated the prevalence of IBS in the Arab world; (ii) studies that were published in English, and (iii) studies that were published between 2019 and 2024. The exclusion criteria for the systematic review included studies that identified the prevalence of IBS but were conducted outside the Arab world.

Data collection process

Database search results were transferred to a reference manager (EndNote 20, Clarivate Plc, Philadelphia, Pennsylvania, United States). At this stage, the duplicates were removed. For screening the potential studies, the EndNote file was transferred to Rayyan, a research collaboration platform [14]. Two independent reviewers were involved in the further process. In the Ryyan software, the blind was turned on to ensure that there was no bias in the selection process. Next, the selection of studies was based on the title and abstract of the studies. Finalized studies were cross-checked by both reviewers and uncommon studies were either excluded or included by discussion among reviewers. In case of any differences in opinion, a third reviewer was involved in the process to finalize the status of inclusion. After that, the detailed data from the searched studies were transferred to Excel (Microsoft Corporation, Redmond, Washington, United States), and notes about the intervention used, participants, and results were obtained.

Results

**Figure 1:**
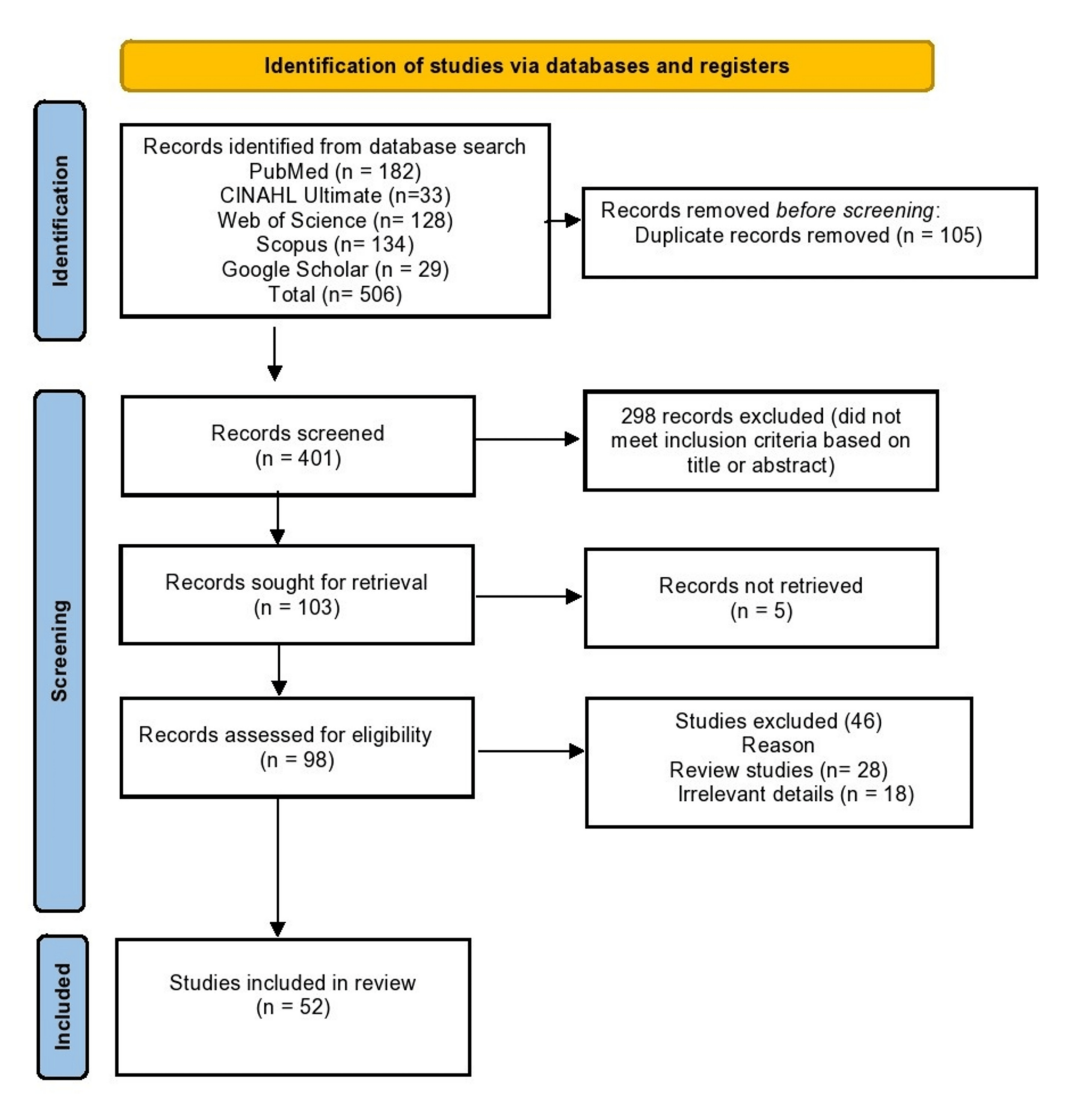
PRISMA flow diagram of the systematic review PRISMA: Preferred Reporting Items for Systematic Reviews and Meta-Analyses

Figure 1 shows the Preferred Reporting Items for Systematic Reviews and Meta-Analyses (PRISMA) flow diagram of the systematic review.

Included Studies

The literature search yielded 506 studies from PubMed (n=182), CINAHL Ultimate (n=33), Scopus (n=134), Web of Science (n=128), and Google Scholar (n=29) (See Appendix B). A total of 105 duplicates were removed prior to the screening process. Out of 401 studies included in the screening process, 298 were removed based on the title and abstract. Finally, after thoroughly screening the studies, only 52 studies met the inclusion criteria for the systematic review.

Study Characteristics

The reviewed characteristics were study design, author year, country of research, number of participants along with their gender, selected population, duration of the study, diagnostic criteria used in the study, and the reported prevalence (Table 1). This systematic review included 52 cross-sectional studies from different countries of the Arab world (Saudi Arabia, Jordan, Egypt, Lebanon, Bahrain, Oman, and Tunisia) cumulating a total of 51,683 participants. All the included studies were cross-sectional studies. The sample sizes within these studies vary from the smallest at 40 [15] to the largest being 14268 [16] participants. Male and female representation across these studies display substantial variations from a full male cohort in the study of Alshahrani et al. [17] to the absence of male participants in Alharbi et al. [18] and Alanzi et al. [19].

The prevalence of IBS among these studies was variable ranging from 7.6% [20] to 54.8% [19]. The average prevalence across these studies is approximately 27%. High prevalence rates of more than 40% were seen among the undergraduate medical students of Saudi Arabia (49.3%) [21], the general population of Saudi Arabia (46.15%) [22], adults of Lebanon (46.8%) [23], adults of Makkah (44.9%) [24], and among the adult citizens of Saudi Arabia (43.6%) [25].

Low prevalence was seen in the adult general population of Saudi Arabia ((7.9%) [26], 8.8% [27], and 11.8% [28]), medical students of Jordan (13%) [29], medical students of Saudi Arabia (12.6% [30] and 14.8% [31,32]) University students of Jordan (16%) [33]. The majority of the studies presented IBS prevalence rates were predominant in females [15,21,23,24,26,28,30,33-46] while two out of 52 studies presented male predominance in IBS prevalence rates [31,47].

Family history was a strong risk factor for IBS in various studies [15-18,24,29,32-35,46,48-50]. Anxiety and depression were also reported as the major contributors [8,16,22,23,27,29,38,41,43,44,46,48-57]. Among the least common risk factors are gastroesophageal reflux diseases [49], low income [26], diabetes mellitus [17], low water intake [28], workload [42,45], and occupation [24]. abdominal pain [15,35]. Other factors that were involved significantly in the high prevalence rate of IBS were food allergy [23,24,35,49,54], smoking [23,27,30,39,43,47-49,57], age [24,29,37,58,59], chronic diseases [18,24,42,48], and stress [8,22,31,32,39,43,49,60].

**Table 1:** Characteristics of the included studies IBS: irritable bowel syndrome

	Design	Country	Number of Participants	Gender	Duration of the Study	Selected Population	Diagnostic Criteria	Prevalence of IBS
Alawi et al. (2024) [34]	Cross-sectional	Bahrain	492	Both	One month	Adults (+18) of Bahrain	Rome IV criteria	18.3%
Zedan et al. (2024) [15]	Cross-sectional	Egypt	40	Both	_	Undergraduate physiotherapy students	Rome IV criteria	25%
ElSharawy et al. (2022) [54]	Cross-sectional	Egypt	182	Both	N/A	Medical students	Rome III criteria	27.5%
Elhosseiny et al. (2019) [46]	Cross-sectional	Egypt	400	Both	October 2018 to February 2019	Medical students	Rome III criteria	31.7%
Abdel-Qader et al. (2024) [57]	Cross-sectional	Jordan	1042	Both	July to September 2023	Adult population	Rome IV criteria	41.7%
Jadallah et al. (2022) [41]	Cross-sectional	Jordan	1135	Both	January to April 2020	1^st^ to 6^th^-year medical students	Rome III criteria	30.9%
Farah et al. (2022) [29]	Cross-sectional	Jordan	585	Both	January to September 2018	Medical students of basic and clinical years	Rome IV criteria	13%
Al-shdaifat et al. (2019) [33]	Cross-sectional	Jordan	163	Both	March 2017	Students of Hashemite University	Rome III criteria	16%
Yazbek et al. (2023) [23]	Cross-sectional	Lebanon	425	Both	June to December 2022	Lebanese adults (+18 years old)	Rome IV criteria	46.8%
AlMutori et al. (2020) [50]	Cross-sectional	Oman	464	Both	June to July 2017	University students	Rome IV criteria	38.9%
Alshaikh et al. (2024) [51]	Cross-sectional analytical	Saudi Arabia	379	Both	November and December 2023	Students of Saudi Arabian Universities	Rome IV questionnaire	31.9 %
Almuzaini et al. (2024) [35]	Cross-sectional descriptive	Saudi Arabia	402	Both	October 2020	Adult residents of the Qassim region	Rome IV criteria	21.4%
Aljahdli et al. (2024) [36]	Cross-sectional web-based survey	Saudi Arabia	1346	Both	February to May 2021	Patients diagnosed with IBS	Rome IV criteria	26.4%
Alhazmi et al. (2024) [52]	Cross-sectional, observational, descriptive	Saudi Arabia	637	Both	_	The general population of Jezan	_	31.08%
Alnasser et al. (2023) [37]	Cross-sectional observational	Saudi Arabia	279	Both	November 2022	Adults with Saudi citizenship	Rome III criteria	17.6%
Hafiz et al. (2023) [24]	Cross-sectional	Saudi Arabia	936	Both	November 2022 to May 2023	Adults of Makkah region (age 25 to 55 years)	Rome IV criteria	44.9%
Alshahrani et al. (2023) [25]	Cross-sectional	Saudi Arabia	1622	Both	April to June 2021	Adult citizens of Saudi Arabia	Rome III criteria	43.6%
Mohammed et al. (2023) [48]	Cross-sectional	Saudi Arabia	600	Both	September 2021 to January 2022	Undergraduate University students	Rome III criteria	20.30%
Agwa et al. (2023) [61]	Cross-sectional	Saudi Arabia	452	Both	July 2023 to July 2023	College students between the ages of 18 to 29 years	Rome IV criteria	36.9%
Alhammadi et al. (2023) [58]	Cross-sectional	Saudi Arabia	683	Both	5 September to 10 October 2022	Population of Asser region	Rome IV criteria	39.97%
Mujamammi et al. (2023) [59]	Cross-sectional and analytical	Saudi Arabia	426	Both	November to December 2020	Students of King Saud University	Rome IV criteria	17.8%
AA et al. (2023) [62]	Cross-sectional	Saudi Arabia	450	Both	October 2021 to March 2022	Secondary school teachers	Rome III criteria	19.4%
Aghamdi et al. (2023) [38]	Observational cross-sectional	Saudi Arabia	402	Both	July 2022 to January 2023	Medical students from several universities	Rome IV criteria	18.7%
Al-Zahrani et al. (2022) [63]	Cross-sectional	Saudi Arabia	303	Both	October and November 2021	Medical students of the University	Rome IV criteria	33%
Alharbi et al. (2022) [8]	Cross-sectional	Saudi Arabia	921	Both	February to April 2022	General population of Makkah city	Rome IV criteria	20.19%
Alfaqih et al. (2022) [31]	Cross-sectional	Saudi Arabia	290	Both	January to March 2022	Medical students	Rome IV criteria	14.8%
Basharat et al. (2022) [39]	Cross-sectional	Saudi Arabia	6300	Both	June to November 2022	General population	Rome IV criteria	23.81%
Alharbi et al. (2022) [18]	Cross-sectional	Saudi Arabia	401	Females	June and July 2021	Secondary school students	Rome IV criteria	21.4%
Alqahtani et al. (2022) [49]	Cross-sectional	Saudi Arabia	1680	Both	June to November 2019	General population	Rome IV criteria	18.2%
Alqumayzi et al. (2022) [40]	Cross-sectional	Saudi Arabia	384	Both	November 2021 to December 2022	Medical students	Rome IV criteria	39.6%
Mirghani et al. (2022) [53]	Cross-sectional	Saudi Arabia	215	Both	June and July 2021	Medical students	Rome III criteria	22.80%
Fadl et al. (2022) [21]	Cross-sectional	Saudi Arabia	300	Both	January to February 2021	Undergraduate medical students	Rome III criteria	49.3%
Abdulrahman et al. (2022) [64]	Cross-sectional	Saudi Arabia	2802	Both	March to June 2021	General population	Rome IV criteria	16.4%
Alreshidi et al. (2022) [60]	Cross-sectional	Saudi Arabia	308	Both	November 2021 to December 2022	Medical students of Hail University	Rome IV criteria	21.5%
Issa et al. (2022) [10]	Cross-sectional	Saudi Arabia	542	Both	August 2021 to July 2022	Young adults of Jeddah	_	18.5%
Selim et al. (2022) [22]	Cross-sectional	Saudi Arabia	806	Both	December 2019 to March 2020	General population	Rome IV criteria	46.15%
Alanzi et al. (2021) [19]	Cross-sectional	Saudi Arabia	230	Females	April to December 2019	High school female teachers	Rome IV criteria	54.8%
Hussein et al. (2021) [26]	Cross-sectional	Saudi Arabia	1319	Both	November 2021 to February 2020	General adult population	Rome IV criteria	7.9%
Basharat (2021) [42]	Cross-sectional	Saudi Arabia	578	Both	_	Teachers working in different schools	Rome Criteria	35.5%
Alshahrani et al. (2020) [17]	Cross-sectional	Saudi Arabia	400	Males	February 2020	Male students of secondary schools	Rome IV criteria	39.80%
Arishi et al. (2020) [43]	Cross-sectional	Saudi Arabia	1554	Both	January to March 2020	Adult general population	Rome IV criteria	16%
Aljammaz et al. (2020) [44]	Cross-sectional	Saudi Arabia	426	Both	March to May 2019	General population	Rome III criteria	30.5%
AlButaysh et al. (2020) [32]	Cross-sectional	Saudi Arabia	290	Both	February to June 2018	Medical students	Rome IV criteria	14.8%
Ahmed et al. (2020) [30]	Cross-sectional	Saudi Arabia	472	Both	October 2019 to January 2020	Medical students	Rome IV criteria	12.6%
Wani et al. (2020) [47]	Cross-sectional	Saudi Arabia	181	Both	January to March 2016	University students	_	29.80%
AlAmeel et al. (2020) [45]	Cross-sectional	Saudi Arabia	594	Both	May and June 2018	Board-certified surgeons and physicians	Rome IV criteria	16.3%
Aljasser et al. (2020) [55]	Cross-sectional	Saudi Arabia	246	Both	_	Medical students	_	35%
Taha et al. (2019) [56]	Cross-sectional	Saudi Arabia	205	Both	_	Primary healthcare workers	Rome IV criteria	16.1%
Hakami et al. (2019) [27]	Cross-sectional	Saudi Arabia	890	Both	March 2017 to May 2018	General population	Rome IV criteria	8.8%
Alharbi et al. (2019) [28]	Cross-sectional	Saudi Arabia	920	Both	October 2018 to February 2019	General population	Rome IV criteria	11.8%
Gallas et al. (2022) [20]	Cross-sectional	Tunisia	343	Both	February to March 2015	Medical students	Rome III criteria	7.6%
Alotaibi et al. (2023) [16]	Cross-sectional observational	United Arab Emirates	14268	Both	2021 to 2022	Graduates and undergraduates of United Arab Emirates University	N/A	39%

Discussion

IBS is a chronic functional bowel disease that is described by altered stool frequency or form associated with abdominal pain and discomfort. Estimation of the worldwide prevalence of IBS is imperative for understanding the burden and distribution of the disease. The incidence and prevalence of IBS have been studied systematically but there are significant variations in the results that may be due to the designs of the study or due to geographical regions. Variations can also occur due to different methods, sampling approaches, questionnaire types, variations due to local factors, and the use of dissimilar diagnostic criteria [65].

This systematic review was aimed at reviewing the prevalence of IBS in the Arab nations. This systematic review was based on 52 studies published from 2019 to 2024 in different Arab countries. The studies included diverse populations ranging from students, medical students, school teachers, adults, and the general population of different countries. All the included studies were cross-sectional and followed Rome III and Rome IV diagnostic criteria. The range of sample size was also vast, ranging from 40 to 14268. The IBS prevalence among the included studies was variable, ranging from 7.6% to 54.8%. The average IBS prevalence across these studies was approximately 27%.

The findings of our study were in line with a recent systematic review conducted for the assessment of the prevalence of IBS in the Kingdom of Saudi Arabia including 20 studies with a total of 1708 participants. This study found that the prevalence rates for IBS in the included studies ranged from 7.9% to 49.3% and the average incidence across these studies was about 24%. The factors highlighted by this study to be significantly related to the high prevalence of IBS were female gender, anxiety, depression, emotional stress, and family history [66]. A similar systematic review and meta-analysis based on 38 studies and 26,567 cumulative participants reported a pooled prevalence of IBS (20.7%) in the Saudi population [9].

Compared to our systematic review, a much lower prevalence has been reported by Oka et al. [65]. Their systematic review assessed the global prevalence of IBS and included 57 studies. The observed prevalence of IBS in studies that used Rome III diagnostic criteria was 9.2% compared with 3.8% who used Rome IV diagnostic criteria. The most commonly reported type of IBS in this systematic review was the mixed type. Moreover, the prevalence of IBS was higher in women as reported in most of the studies in our review. The prevalence was variable in different countries. This variability in prevalence persisted even when similar diagnostic criteria were used with identical procedures. However, this systematic review indicated a substantially low incidence with Rome IV criteria indicating that more restrictive standards can be less appropriate for population-based prevalence surveys [65]. A population-based study conducted in Iran found a very low 1.1% prevalence of IBS. The factors that were associated significantly were older age and marital status like some of the reported studies in the current review [67]. However, this study was published in 2009. This also indicates an increase in IBS prevalence in the region.

As reported in the present systematic review, the higher prevalence of IBS in women has been reported previously as well. A systematic review and meta-analysis by Lovell et al. reported an odds ratio of 1.67 in women compared to men regarding IBS [68]. Male predominance was also observed in two of the included studies in this systematic review [31,47]. Most of the global prevalence-reported studies marked a female predominance for IBS. It is believed sex hormones may promote gender differences by affecting stress hormones, immune response, gut-brain interactions, intestinal barrier functionality, and gut microbiome [69]. In their systematic review, Saito et al. found that the incidence of IBS in North America ranges from 3% to 20% [70]. The prevalence estimates ranged from 10% to 15% in most of the included studies. In their meta-analysis, Lovell et al. used Rome I and II criteria and found that the prevalence values were 8.8% and 9.4% respectively. Like other studies, this prevalence was higher in females than males. Also, the reported prevalence was lower in older adults and high in younger adults [71].

The most common risk factors found, in most of the studies included in the current systematic review, to be significantly associated with high prevalence rates of IBS were similar to the findings of the previous studies specifically designed to find the risk factors for IBS [72,73]. Depression and anxiety were considered to be the main culprits behind the progression of IBS. This is also evident from our study as several of the included studies found a significant association of depression and anxiety with IBS. On the other hand, IBS can also result in depression and anxiety in individuals. This was evidenced by Zamani et al. in their systematic review [74]. They found that the prevalence rates of anxiety disorders and symptoms in individuals suffering from IBS were 39.1% and 23%, respectively. Depressive disorders and symptoms were calculated to be 28.8% and 23.3%, respectively. These prevalence rates were an indication that patients with IBS have 3 increased odds of depression and anxiety compared to healthy subjects.

The majority of studies included in this systematic review included students of universities. This can be due to the limitation of the methodology as the majority of the studies used a cross-sectional study design. Furthermore, the high prevalence of IBS in students as reported in most studies can be due to stress-related factors. A systematic review that assessed the prevalence of IBS in medical students reported that IBS incidence ranges from 9.3% to 35.5% [75]. The prime factor for this high prevalence was a stressful environment in addition to the female gender, emotional disorders, anxiety, dietary habits, poor sleep, depression, and poor quality of sleep. Another systematic review was conducted for the assessment of the prevalence of IBS in Chinese university students [76]. This systematic review reported a pooled prevalence of IBS to be 11.89%. However, this prevalence was variable among the different diagnostic criteria used in the study. The highest prevalence of IBS was 17.66% in North China and the lowest was 3.18% in South China. Similar to the current review, this study also indicated that anxiety, depression, gender, and smoking behaviors were significantly associated with the prevalence of IBS. This study did not find any definite link between caffeine to IBS, but there was a marginal rise in the risk linked with its use [77].

## Conclusions

The prevalence of IBS shows significant variability across different populations and countries. The female predominance in IBS prevalence was a consistent observation across a majority of the studies reviewed. Several risk factors identified included gastroesophageal reflux disease, low income, diabetes mellitus, low water intake, workload, occupation, food allergy, smoking, age, chronic diseases, and stress.

## Appendices

Appendix A

**Table 2:** Combination of search terms used for literature search TI: title; AB: abstract; MeSH: medical subject headings

Search terms used		
#1 Distribution [TI/AB]	#14 Middle East [MeSH]	#27 Palestine [TI/AB]
#2 Epidemiology [TI/AB]	#15 Middle East [TI/AB]	#28 Qatar [TI/AB]
#3 Incidence [TI/AB]	#16 Bahrain [TI/AB]	#29 Saudi Arabia [TI/AB]
#4 Prevalence [TI/AB]	#17 Djibouti [TI/AB]	#30 Somalia [TI/AB]
#5 OR/1-4	#18 Egypt [TI/AB]	#31 Sudan [TI/AB]
#6 Irritable bowel syndrome [TI/AB]	#19 Iran [TI/AB]	#32 Syria [TI/AB]
#7 IBS [TI/AB]	#20 Iraq [TI/AB]	#33 Tunisia [TI/AB]
#8 Spastic Colon [TI/AB]	#21 Jordan [TI/AB]	#34 United Arab Emirates [TI/AB]
#9 Mucous Colitis [TI/AB]	#22 Kuwait [TI/AB]	#35 Yemen [TI/AB]
#10 Spastic Bowel syndrome [TI/AB]	#23 Lebanon [TI/AB]	#36 Arab world [TI/AB]
#11 Irritable colon [TI/AB]	#24 Libya [TI/AB]	#37 OR/14-36
#12 Functional Bowel Disease [TI/AB]	#25 Morocco [TI/AB]	#38 5 AND 13 AND 37
#13 OR/6-12	#26 Oman [TI/AB]	

Appendix B

**Table 3:** Search results for the terms used in data search

Key Variable	Sub terms	Search options	PubMed	CINAHL ultimate	Web of Science	Scopus
1. Prevelance	1.1 Distribution	Title/Abstract	1,022,446	92464	3316754	4467525
	1.2 Epidemiology	Title/Abstract	271,132	43842	189400	243619
	1.3 Incidence	Title/Abstract	964,325	211246	1009927	1331650
	1.4 Prevalence	Title/Abstract	844,778	237884	874621	1050688
(((Distribution[Title/Abstract]) OR (Epidemiology[Title/Abstract])) OR (Incidence[Title/Abstract])) OR (Prevalence [Title/Abstract])			2,849,135	536568	5161383	6791471
2. Irritable bowel syndrome	1.1 Irritable bowel syndrome	Title/Abstract	17,118	4331	19333	20125
	1.2 IBS	Title/Abstract	12,183	2807	15830	17363
	1.3 Spastic Colon	Title/Abstract	63	10	109	240
	1.4 Mucous Colitis	Title/Abstract	26	5	311	571
	1.5 Spastic Bowel syndrome	Title/Abstract	51	1	29	81
	1.6 Irritable colon	Title/Abstract	451	40	1562	2362
	1.7 Functional Bowel Disease	Title/Abstract	178	142	5509	7092
((((((Irritable bowel syndrome[Title/Abstract]) OR (IBS[Title/Abstract])) OR (Spastic Colon [Title/Abstract])) OR (Mucous Colitis [Title/Abstract])) OR (Spastic Bowel syndrome [Title/Abstract])) OR (Irritable colon [Title/Abstract])) OR (Functional Bowel Disease[Title/Abstract])			20,152	5100	30355	32919
3. Arab world	3.1 Middle East	Mesh	168,354			
	3.2 Middle East	Title/Abstract	17,997	4186	59290	90409
	3.3 Bahrain	Title/Abstract	1452	124	3146	6559
	3.4 Djibouti	Title/Abstract	481	71	865	1351
	3.5 Egypt	Title/Abstract	19894	3211	54359	77453
	3.6 Iran	Title/Abstract	57695	21468	114646	165955
	3.7 Iraq	Title/Abstract	9308	4168	25634	50399
	3.8 Jordan	Title/Abstract	9546	3568	33377	46696
	3.9 Kuwait	Title/Abstract	4651	1200	10184	16871
	3.10 Lebanon	Title/Abstract	6775	2174	13355	1800
	3.11 Libya	Title/Abstract	1655	327	5883	10035
	3.12 Morocco	Title/Abstract	8061	1167	26308	36707
	3.13 Oman	Title/Abstract	4332	1123	11612	17989
	3.14 Palestine	Title/Abstract	2389	930	12081	15445
	3.15 Qatar	Title/Abstract	3695	1222	7082	12262
	3.16 Saudi Arabia	Title/Abstract	29932	5936	49061	62848
	3.17 Somalia	Title/Abstract	1975	572	4267	6711
	3. 18 Sudan	Title/Abstract	10207	1600	19969	26167
	3.19 Syria	Title/Abstract	3261	913	12267	18235
	3.20 Tunisia	Title/Abstract	8671	897	20513	26950
	3.21 United Arab Emirates	Title/Abstract	4187	1230	9101	14583
	3.22 Yemen	Title/Abstract	2456	634	5531	8416
	3.23 Arab world	Title/Abstract	835	325	6769	12349
(((((((((((((((((((((("Middle East"[Mesh]) OR (Middle East[Title/Abstract])) OR (Bahrain[Title/Abstract])) OR (Djibouti[Title/Abstract])) OR (Egypt[Title/Abstract])) OR (Iran[Title/Abstract])) OR (Iraq[Title/Abstract])) OR (Jordan[Title/Abstract])) OR (Kuwait[Title/Abstract])) OR (Lebanon[Title/Abstract])) OR (Libya[Title/Abstract])) OR (Morocco[Title/Abstract])) OR (Oman[Title/Abstract])) OR (Palestine[Title/Abstract])) OR (Qatar[Title/Abstract])) OR (Saudi Arabia[Title/Abstract])) OR (Somalia[Title/Abstract])) OR (Sudan[Title/Abstract])) OR (Syria[Title/Abstract])) OR (Tunisia[Title/Abstract])) OR (United Arab Emirates[Title/Abstract])) OR (Yemen[Title/Abstract])) OR (Arab world[Title/Abstract])			291220	53012	458862	654050
(((((((((((((((((((((((("Middle East"[Mesh]) OR (Middle East[Title/Abstract])) OR (Bahrain[Title/Abstract])) OR (Djibouti[Title/Abstract])) OR (Egypt[Title/Abstract])) OR (Iran[Title/Abstract])) OR (Iraq[Title/Abstract])) OR (Jordan[Title/Abstract])) OR (Kuwait[Title/Abstract])) OR (Lebanon[Title/Abstract])) OR (Libya[Title/Abstract])) OR (Morocco[Title/Abstract])) OR (Oman[Title/Abstract])) OR (Palestine[Title/Abstract])) OR (Qatar[Title/Abstract])) OR (Saudi Arabia[Title/Abstract])) OR (Somalia[Title/Abstract])) OR (Sudan[Title/Abstract])) OR (Syria[Title/Abstract])) OR (Tunisia[Title/Abstract])) OR (United Arab Emirates[Title/Abstract])) OR (Yemen[Title/Abstract])) OR (Arab world[Title/Abstract])) AND (((((((Irritable bowel syndrome[Title/Abstract]) OR (IBS[Title/Abstract])) OR (Spastic Colon [Title/Abstract])) OR (Mucous Colitis [Title/Abstract])) OR (Spastic Bowel syndrome [Title/Abstract])) OR (Irritable colon [Title/Abstract])) OR (Functional Bowel Disease[Title/Abstract]))) AND ((((Distribution[Title/Abstract]) OR (Epidemiology[Title/Abstract])) OR (Incidence[Title/Abstract])) OR (Prevalence [Title/Abstract]))			182	33	128	134
